# Feature Extraction of Electronic Nose Signals Using QPSO-Based Multiple KFDA Signal Processing

**DOI:** 10.3390/s18020388

**Published:** 2018-01-29

**Authors:** Tailai Wen, Jia Yan, Daoyu Huang, Kun Lu, Changjian Deng, Tanyue Zeng, Song Yu, Zhiyi He

**Affiliations:** 1 College of Electronic and Information Engineering, Southwest University, Chongqing 400715, China; wtl980059723@email.swu.edu.cn (T.W.); huiyuancream@email.swu.edu.cn (D.H.); deng150911@email.swu.edu.cn (C.D.); zty172623471@email.swu.edu.cn (T.Z.); s0531@email.swu.edu.cn (S.Y.); hzy563255@email.swu.edu.cn (Z.H.); 2 Chongqing Key Laboratory of Nonlinear Circuits and Intelligent Information Processing, Chongqing 400715, China; 3 High Tech Department, China International Engineering Consulting Corporation, Beijing 100048, China; lvk@ciecc.com.cn

**Keywords:** electronic nose, feature extraction, multiple kernel learning, weighted kernels Fisher discriminant analysis, classification

## Abstract

The aim of this research was to enhance the classification accuracy of an electronic nose (E-nose) in different detecting applications. During the learning process of the E-nose to predict the types of different odors, the prediction accuracy was not quite satisfying because the raw features extracted from sensors’ responses were regarded as the input of a classifier without any feature extraction processing. Therefore, in order to obtain more useful information and improve the E-nose’s classification accuracy, in this paper, a Weighted Kernels Fisher Discriminant Analysis (WKFDA) combined with Quantum-behaved Particle Swarm Optimization (QPSO), i.e., QWKFDA, was presented to reprocess the original feature matrix. In addition, we have also compared the proposed method with quite a few previously existing ones including Principal Component Analysis (PCA), Locality Preserving Projections (LPP), Fisher Discriminant Analysis (FDA) and Kernels Fisher Discriminant Analysis (KFDA). Experimental results proved that QWKFDA is an effective feature extraction method for E-nose in predicting the types of wound infection and inflammable gases, which shared much higher classification accuracy than those of the contrast methods.

## 1. Introduction

An electronic nose (E-nose) is an expert system composed of a gas sensor array with partial specificity and an artificial intelligence algorithm, which is a sort of artificial intelligent system due to its ability to imitate the olfaction system of humans or mammals and then recognizing the odorant gases. During the past decades, it plays a constantly crucial role in a multitude of fields by detecting the general-purpose vapor chemicals such as disease diagnosis [1,2], food industry [3], volatile organic compounds (VOCs) detection [4], inflammable gases detection [5], etc. Compared with traditional detection technique, the E-nose has advantages of non-invasion, real-time, convenience and high efficiency.

Researchers have contributed a lot to improving the feature extraction methods of the E-nose signals in the past decade due to the critical role of the feature extraction for the prediction of the E-nose. Based on the original response of sensors, the conventional feature extraction methods, such as Principal Component Analysis (PCA) [6] and Fisher Discriminant Analysis (FDA) [7] are promising in finding and keeping the linear structure of data, but have little to do with the situation of E-nose because of the non-linear projection of the chemical sensors between a wide concentration range of odors and the output responses. Consequently, Kernel method [8,9] coupled with Fisher Discriminant Analysis (KFDA) is presented to deal with the nonlinear problem which is the explicit weakness of FDA. With the help of kernel function, KFDA can map the input vectors to high-dimensional feature space, in which the classical FDA is employed to analyze the feature information of data. KFDA can provide an easier and more effective way to extend and generalize FDA for nonlinear discriminant analysis. In the past few years, KFDA has been introduced to a sea of fields. Its performance is influenced strongly by the kernel function, and lots of key information may be ignored or lost during the transformation. To solve this problem, it becomes more accepted nowadays to construct a better kernel from a series of kernels, called multiple kernels learning (MKL) [10,11], which can obtain better mapping performance and possess more optimal performance than any single kernel. More recently, MKL has been utilized gradually in E-nose signal processing and has shown favorable and promising performance in different applications [12,13].

The employment of MKL for KFDA has received much interest in the literature. Tsuda et al. considered both the linear mixture and the nonlinear mixture of kernel matrices and optimized the kernel combination weights to minimize the cross-validation error for KFDA [14]. Scores of other research work have also introduced multiple kernel FDA (MK-FDA) by constructing an optimized linear combination of several base kernels under a specific constraint for mixing weights [15,16,17,18,19,20,21,22]. Fung et al. proposed a fast-iterative algorithm to find an appropriate linear combination of heterogeneous kernels for KFDA with nonnegativity constraints [15]. In the work of Kim et al. and Ye et al. [16,17], the kernel weights are regularized under the ${ℒ}_{1}$ norm constraint. However, as pointed out by Yan et al. [18,19], this kind of MK-FDA is apt to produce sparse selection results, resulting in information loss. To remedy this, Yan et al. extended the work of Kim et al. and Ye et al. to a general ${ℒ}_{p}$ norm regularization on the kernel weights by incorporating some techniques in non-sparse MKL. In addition, Liu et al. also provided an iterative scheme for weight optimization in the MK-FDA formulation [20]. Wang et al. [21], however, utilized MKL to enhance the learning performance of local Fisher discriminant analysis (LFDA). The proposed multiple kernel local Fisher discriminant analysis (MKLFDA) can attain maximum discrimination performance in dimensionality reduction. Moreover, by virtue of MKL, multiple image features extracted from different descriptors can be effectively utilized and the algorithm can handle nonlinear dimensionality reduction problems. More recently, Liu et al. constructed multiple data-dependent kernel (MDK) by virtue of combining several base kernels with a data-dependent kernel constraint on their weights for the termed multiple data-dependent kernel Fisher discriminant analysis (MDKFDA) [22]. 

As we can see, formulations of different MK-FDAs usually concentrate on effectively solving optimization problems of kernel combination coefficients under certain constraints. For instance, Fung et al. sought the optimal linear combination of kernels by incorporating them and the KFDA problem into a quadratic optimization problem with nonnegativity constraints [15]. Kim et al. utilized the semidefinite program (SDP) formulation to obtain the optimal multiple kernel [16]. However, all research lacks consideration of the effects of numbers and types of base kernel constituent, which are usually predetermined arbitrarily but actually pertinent to the algorithm performance. Moreover, all the algorithms specify the kernel parameters of the base kernels as certain specific values and do not optimize them, which may strongly influence the spatial distribution of the data in the high-dimensional feature space. Consequently, the numbers and types of base kernels should be carefully discussed and an effective intelligence optimization algorithm should be incorporated for kernel parameter optimization. On the other hand, the majority of applications of the proposed algorithm are aimed to tackle the classification problem while some recent work has explored the potential of the MKFDA in the feature extraction field [20,21,22]. Furthermore, from a practical point of view, little attention has been devoted to utilizing multiple kernels of FDA in the E-nose signal processing.

The purpose of this paper is to present a weighted KFDA (WKFDA) combined with Quantum-behaved Particle Swarm Optimization (QPSO), i.e., QWKFDA, which incorporates KFDA and multiple kernels methodology to capture more information of E-nose data and test the feasibility of the multiple kernels FDA in processing the E-nose original feature matrix and obtaining a new feature matrix with more useful information. It is hoped that the question of feature extraction will be resolved with our proposed approach. Different from the previous multiple KFDA, our method emphasizes the effect of the number and types of base kernels. In addition, an intelligence optimization algorithm is used for the optimization of the combination coefficients of base kernels (weights), which can be regarded as the contribution of each base kernel, and the kernel parameters of base kernels, which have a strong impact on the spatial distribution of the data in the implicitly defined feature space.

The rest of the paper is organized as follows. In Section 2, we will give the whole derivation process of QWKFDA. Two different E-nose datasets used in our research are introduced in Section 3. In Section 4, all considered methods will be used to deal with the original feature matrix of the two datasets, and the classification results will be presented and analyzed subsequently. In Section 5, we will draw our conclusions.

## 2. Methodology

### 2.1. Review of Kernel Fisher Discriminant Analysis

FDA can find a linear transformation which maximizes the between-class scatter and minimizes the within-class scatter to achieve the maximum class separability. While in KFDA, the kernel mapping can allow the construction of nonlinear decision function in the input space, which is equivalent to the construction of linear decision function in the feature space. Like FDA, the purpose of KFDA is also to maximize the between-class scatter and minimize the within-class scatter.

Suppose that the dimensionality of original sample space is n, the number of the total classes is C, the total original data is $X=\{X^{1},X^{2},\ldots ,X^{C}\}$, the $X^{i}=\{x_{1}^{i},x_{2}^{i},\cdots ,x_{N_{i}}^{i}\}$ containing $N_{i}$ samples of i-th (i=1,2,⋯, C) class is the subsets of X and N is the total number of points in **X**, and then $N=\sum_{i=1}^{C}N_{i}$.

Suppose the input space is mapped into a higher (possibly infinite) dimensional feature space through a nonlinear mapping function ϕ(⋅):

(1) $$\begin{array}{c} \phi :\;x\in R^{n}\to \phi (x)\in ℱ\\ ℒ\to ℱ \end{array}$$

The mapping of sample $x_{j}^{i}$ in feature space is noted as $\phi (x_{j}^{i})$. Then we can obtain the *i*-th mapped class and the total mapped sample sets are given by:

(2) $$\phi (X^{i})=\{\phi (x_{1}^{i}),\phi (x_{2}^{i}),\cdots ,\phi (x_{N_{i}}^{i})\}.$$

(3) $$\phi (X)=\{\phi (x_{1}^{1}),\cdots ,\phi (x_{N_{1}}^{1}),\cdots ,\phi (x_{1}^{C}),\cdots ,\phi (x_{N_{C}}^{C})\}.$$

For convenience, it is assumed that the points are centered in ℱ. Let $S_{B}$ represent the between-class scatter in the feature space:

(4) $$S_{B}=\frac{1}{N}\sum_{i=1}^{C}N_{i}m_{i}m_{i}^{T},$$

where $m_{i}=\frac{1}{N_{i}}\sum_{j=1}^{N_{i}}\phi (x_{j}^{i})$ is the mean value of the mapped points of class i.

Let $S_{W}$ be the within-class scatter matrix in the feature space ℱ:

(5) $$S_{W}=\frac{1}{N}\sum_{i=1}^{C}\sum_{j=1}^{N_{i}}\left(\phi (x_{j}^{i})-m_{i}\right){\left(\phi (x_{j}^{i})-m_{i}\right)}^{T}.$$

For the common FDA [7], the classical criteria for class separability is defined by the quotient between the between-classes scatter and the within-class scatter. It means maximizing the between-class scatter and minimizing the within-class scatter. This maximization problem is equivalent to solving Equation (6): find eigenvalues λ and eigenvectors w. The maximum is reached for the largest eigenvalue in Equation (6).

(6) $$\lambda S_{W}w=S_{B}w.$$

Because the eigenvectors are linear combination of elements in space, there exist coefficients $\alpha_{q}^{p}(p=1,2,\ldots ,C;q=1,2,\ldots ,N_{p})$ such that

(7) $$w=\sum_{p=1}^{C}\sum_{q=1}^{N_{p}}\alpha_{q}^{p}\phi (x_{q}^{p})=\phi \left(X\right)\alpha ,$$

where $\alpha ={\left[\alpha_{1}^{1},\alpha_{2}^{1},\cdots ,\alpha_{N_{1}}^{1},\cdots ,\alpha_{1}^{C},\alpha_{2}^{C},\cdots ,\alpha_{N_{C}}^{C},\right]}^{T}$.

We define a kernel function k as:

(8) $$k_{lk}=k(x_{l},x_{k})=\phi^{T}(x_{l})\phi (x_{k}),$$

and then a KFDA can be constructed using the kernel function exclusively, without having to consider the mapping explicitly.

For given classes p and q, we express this kernel function by:

(9) $${\left(k_{lk}\right)}_{pq}=\phi^{T}(x_{l}^{p})\phi (x_{k}^{q}).$$

Let **K** be a N×N matrix, and $K={\left(K_{pq}\right)}_{\begin{array}{c} p=1,2,\ldots ,C\\ q=1,2,\ldots ,C \end{array}}$, where $\left(K_{pq}\right)$ is a matrix composed of inner product in the feature space:

(10) $$K={\left(K_{pq}\right)}_{\begin{array}{c} p=1,2,\ldots ,C\\ q=1,2,\ldots ,C \end{array}},K_{pq}={\left(k_{lk}\right)}_{\begin{array}{c} l=1,2,\ldots ,N_{p}\\ k=1,2,\ldots ,N_{q} \end{array}},$$

where $K_{pq}$ is a $\left(N_{p}\times N_{q}\right)$ matrix and K is symmetric matrix such that $K_{pq}^{T}=K_{pq}$.

Thus Equation (6) can be rewritten as

(11) (KBK)α=λ(KK)α,

where $K=\phi {\left(X\right)}^{T}\phi (X)$, $B={\left[\begin{array}{llll} B_{1} & & & 0\\ & B_{2} & & \\ & & \ddots & \\ 0 & & & B_{C} \end{array}\right]}_{N\times N}$, and $B_{i}=\frac{1}{N_{i}}{\left[1\right]}_{N_{i}\times N_{i}}$ is a $N_{i}\times N_{i}$ matrix with terms all equal to $\frac{1}{N_{i}}$. It means affording the inner products of pattern vectors in high-dimensional feature space ℱ by a kernel function.

Consequently Equation (6) is equal to finding the maximum of Equation (12)

(12) $$\lambda =\frac{\alpha^{T}KBK\alpha }{\alpha^{T}KK\alpha }.$$

By solving Equation (12), the coefficients α can be computed, and finally we get eigenvectors w, which are ordered according to their eigenvalues. Define $W=[w_{1},\ldots ,w_{C-1}]$, and eigenvectors $w_{i}(i=1,\cdots ,C-1)$ are all normalized. Let $Y=[y_{1},y_{2},\ldots ,y_{N}]$, which is the projections of ϕ(X) onto the eigenvectors, be the objective feature matrix corresponding to input matrix ϕ(X), so

(13) $$Y=W^{T}\phi (X)=A^{T}\phi {\left(X\right)}^{T}\phi (X)=A^{T}K,$$

where $A=[\alpha_{1},\alpha_{2},\ldots ,\alpha_{C-1}]$. For a new point z in input space, its corresponding projection onto W in high-dimensional feature space ℱ is

(14) $$g=W^{T}\phi (z)={\left[\phi (X)A\right]}^{T}\phi (z)\;=A^{T}\phi {\left(X\right)}^{T}\phi (z)=A^{T}\left[\begin{array}{c} \phi {\left(x_{1}\right)}^{T}\phi (z)\\ \vdots \\ \phi {\left(x_{N}\right)}^{T}\phi (z) \end{array}\right]=A^{T}\left[\begin{array}{c} k(x_{1},z)\\ \vdots \\ k(x_{N},z) \end{array}\right].$$

### 2.2. Properties of Mercer’s Kernels

In the context of KFDA, one can use any kernel function to fulfill Mercer’s condition [23], which can be stated formally in the following theorem.

*Theorem: Mercer’s Theorem.* Let X be a compact subset of $R^{n}$, $f\in L^{2}(X)$, k:X×X→R be a symmetric function, then k is a Mercer’s kernel equivalent to the conditions outlined in formula (15):

(15) $$\int_{X\times X}k(x,z)f(x)f(z)dxdz\geq 0.$$

It is also equivalent to that the kernel matrix K, formed by restricting k to any finite subset of X, is positive semi-definite, i.e., having no negative eigenvalues. In addition, paramount properties of Mercer’s kernels can be derived from the fact that they are positive-definite matrices, as follows.

*Proposition: Properties of Mercer’s Kernels.* Let $k_{1}$ and $k_{2}$ be valid Mercer’s kernels over X×X, with $x_{i}\in X\subseteq R^{n}$, and $a\in R^{+}$. Then the following functions are valid kernels:

(1) $k(x_{i},x_{j})=ak_{1}(x_{i},x_{j})$;
(2) $k(x_{i},x_{j})=k_{1}(x_{i},x_{j})+k_{2}(x_{i},x_{j})$.

It is worth noting that the size of the training kernel matrix is N×N and each position (i,j) of matrix ${\left(K\right)}_{ij}$ contains the similarity among all possible pairs of training samples ($x_{i}$ and $x_{j}$) measured with a suitable kernel function fulfilling Mercer’s conditions. Quite a few popular kernels include polynomial kernel function $k(x_{i},x_{j})={\left(c_{0}\langle x_{i},x_{j}\rangle +c_{1}\right)}^{d}$, Gaussian kernel function $k(x_{i},x_{j})=\exp(-{\left\|x_{i}-x_{j}\right\|}^{2}/\sigma^{2})$, Sigmoid kernel function $k(x_{i},x_{j})=\tanh(\beta_{0}\langle x_{i},x_{j}\rangle +\beta_{1})$. The (distance or similarity) matrix is precomputed at the beginning of FDA procedure, and thus, one usually works with the transformed input data K rather than the original input space samples $x_{i}$. This fact allows us to easily unify the positive definite kernel matrices taking advantage of the properties in Proposition, as will be shown in the next section.

### 2.3. QPSO-Based Weighted Kernel Fisher Discriminant Analysis Model

In KFDA, a multitude of the model’s characteristics in Equation (14) are determined by the type of kernel matrix K. The characterization of a kernel is done by means of the Mercer’s theorem and each kernel has its own advantages and disadvantages. However, the mixture of kernels with different characteristics may integrate the advantages of various kernels, obtain better mapping performance and enjoy more optimal performance than any single kernel. Meanwhile, quintessential learning problems often involve multiple or heterogeneous data and multiple kernel methodology can provide better flexibility. Moreover, multiple kernel methodology can be used as an artful approach to explain the learning result and obtain deeper understanding of problems. Accordingly, we propose a QPSO-based weighted kernel Fisher discriminant analysis to extract features of wound infection data and reprocess the original feature matrix.

Let $k_{1}$ and $k_{2}$ be kernels over X×X, $X\subseteq R^{n}$, and from (15) we can write

(16) $$\begin{array}{l} \int_{X\times X}k_{1}(x,z)f(x)f(z)dxdz\geq 0\\ \int_{X\times X}k_{2}(x,z)f(x)f(z)dxdz\geq 0 \end{array}.$$

Suppose $p_{1},p_{2}\in R^{+}$, by exploiting properties (1) and (2) in Proposition, we can obtain

(17) $$\int_{X\times X}k_{c}(x,z)f(x)f(z)dxdz\geq 0,$$

where $k_{c}(x,z)=p_{1}k_{1}(x,z)+p_{2}k_{2}(x,z)$.

According to (15), the composite kernel function $k_{c}$ is a Mercer’s kernel, and thus a weighted kernel that will balance the different base kernels in (18) can be created as follows:

(18) $$k(x_{i},x_{j})=\sum_{m=1}^{n}p_{m}k_{m}(x_{i},x_{j}),$$

where *n* is the number of the base kernels, $k_{m}(m=1,\cdots ,n)\;$ is the base kernel with different values of kernel parameters and $p_{m}(m=1,\cdots ,n)\;$ is a positive real-valued parameter, which is tuned in the training process and constitutes a tradeoff among different base kernels to map a given sample. For better interpretation, the $p_{m}$ can, therefore, be regarded as measures of relative importance (or relative contribution) of one base kernel with respect to the others considered in the composition. Now we can see the composite kernel is a weighted summation of the base kernels with $p_{m}$ as the corresponding weighting coefficient for each one. This weighted kernel allows us to introduce a priori knowledge in the feature extraction for each class, and also allows us to extract more information from the best tuned parameter. In this paper, we employ various kernels with different parameters as base kernel function of WKFDA.

Considering the influence of the kernel parameters and the weighting coefficient on the results, various intelligent optimization algorithms have been applied for the selection of the parameters and devoted to E-nose pattern recognition. In order to make WKFDA obtain the best performance, due to the superiority in the complexity and especially the efficiency of QPSO [24,25,26], it is leveraged for the optimization of the weighting parameters $p_{m}$ in Equation (18) and the model parameters of the three different base kernels to construct of a weighted multiple kernel and then perform WKFDA shown in Equation (14), which is named QPSO-based weighted kernel Fisher discriminant analysis (QWKFDA).

## 3. Description of Experimental Data

In this section, we will employ proposed QWKFDA algorithms on E-nose data. In order to verify the effectiveness of the QWKFDA algorithms, two E-nose datasets which were derived from different applications of E-nose are exploited and studied in this paper.

### 3.1. Dataset I

**Figure 1:**
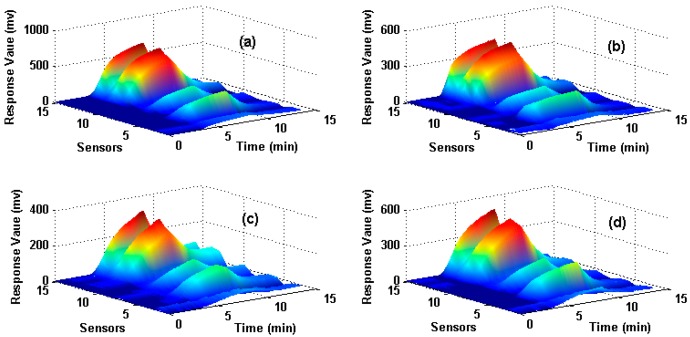
Response process of sensors on four different types of wounds Subfigures (**a**–**d**) correspond to the uninfected wound, wounds infected by *S. aureus*, *E. coli* and *P. aeruginosa* respectively.

The dataset is for wound infection detection using a home-made E-nose, which was presented firstly in [27]. It contains 80 measurements (observations) from the E-nose system with 15 gas sensors exposed to four different types of wound headspaces. The four types of wounds are uninfected wound and wounds infected with Pseudomonas aeruginosa, Escherichia coli, and Staphylococcus aureus respectively. The maximum value is extracted from each sensor response to construct the original feature matrix and thus a 15-dimensional feature vector (1 feature × 15 sensors) for each observation is obtained. In total, 80 observations that are collected from four types of wounds (20 observations for each type) are included in the data set and the data structure of the original feature matrix is 80 × 15 (row: 80 observations, column: 15-dimensional features). Figure 1 illustrates the sensor responses process of four measurements when they are exposed to four different target wounds, where *X*-axes denote the response time of the sensors, *Y*-axes denote the number of the sensors and *Z*-axes denote the response value. Figure 1a–d corresponds to uninfected, infected by *S. aureus*, *E. coli* and *P. aeruginosa* wounds respectively. Hold-out technique is adapted to train and test the SVM classifier. We randomly select 50% samples to establish the training set, and the rest 50% samples are used as the test set. The same train and test procedure is implemented 10 times repeatedly for each method and the average classification accuracy of the test set is used to evaluate each feature extraction method.

### 3.2. Dataset II

**Figure 2:**
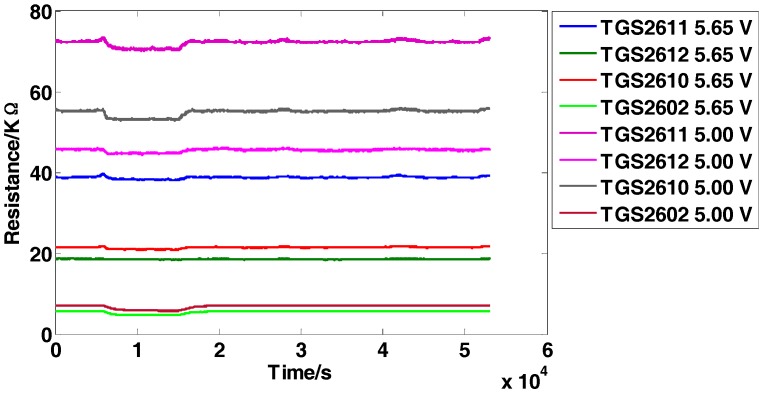
Response curves of the sensors on carbon monoxide with the concentration of 25 ppm.

The dataset is for the detection of four inflammable gases (ethylene, ethanol, carbon monoxide, and methane) using an E-nose, which was presented firstly in [5]. It contains 640 observations from 5 independent batches (E-noses) following the same system design and implementation. Each batch consists of 8 MOS sensors from four different models (TGS2611, TGS2612, TGS2610, TGS2602). Two repetitions of the same sensor model are used but operate at two different voltages (5.00 V and 5.65 V, respectively) induced in the heater. The minimum value is extracted from each sensor response to construct the original feature matrix and thus an 8-dimensional feature vector (1 feature × 8 sensors) for each observation is obtained. The five independent batches are tested several times (a total of 16 days) over a 22-day period and no tests are conducted on the 5th, 6th, 12th, 13th, 19th and 20th days. Each day, one single batch is applied to test the four types of gases with 10 different concentration levels and obtains 40 samples in total, and finally 640 observations (4 gases × 10 concentrations × 16 days) are obtained. The data structure of the original feature matrix is 640 × 8 (row: 640 observations, column: 8-dimensional features). Figure 2 manifests the representative response curves of batch 1 exposed to carbon monoxide with the concentration of 25 ppm. For proving the effectiveness of the proposed methods, the experimental setting is given as follows: Take batch 1 as fixed training set and tested on batch M (M = 2,…,5).

## 4. Results and Discussion

The QWKFDA will be used to extract the feature information of the two datasets as the proposed method. PCA, LPP [28], FDA and KFDA are employed to act as controlled trials to demonstrate the validity of the proposed method. Meanwhile, FDA and KFDA are also employed to prove whether QWKFDA is able to improve the performance in dealing with the nonlinear problem of chemical sensor array. 

### 4.1. Results of Dataset I

**Figure 3:**
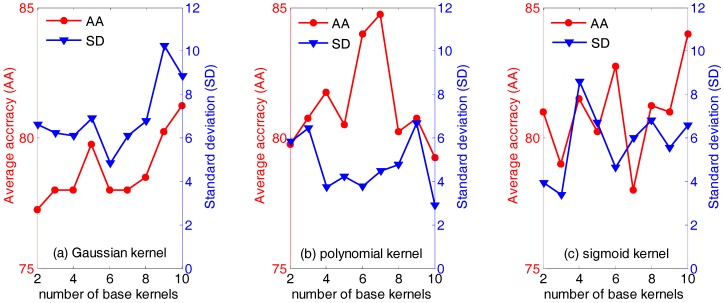
The performance of weighted kernels Fisher discriminant analysis combined with quantum-behaved particle swarm optimization (QWKFDA) using different kernels with the number of base kernels from 2 to 10.

First of all, the influence of the type of and the number of base kernels (the parameter *n* in Equation (18)) are evaluated. Figure 3 below displays the average accuracies (AA) and the standard deviation (SD) of QWKFDA with different kernels, in which the number of base kernels varies from 2 to 10. It is obvious that the average classification accuracy and standard deviation vary with the different number of base kernels. Generally, for the polynomial kernel, it can obtain not only higher average classification accuracy but also lower standard deviation, compared with the Gaussian kernel and sigmoid kernel. Especially, the highest average classification accuracy (84.75%) is obtained when the number of base kernels is 7. For the Gaussian kernel, it is evident that the average classification accuracies are generally below 80% no matter how many base kernels are used to construct the weighted kernel function, except *n* equals 9 or 10. QWKFDA using the Gaussian kernel presents the lowest average classification accuracy and the highest standard deviation in general. It means that QWKFDA using the Gaussian kernel is easily influenced by the distribution of the training data and cannot obtain a robust classifier for different training set. On the contrary, QWKFDA using the polynomial kernel can obtain more robust classifier model. Thus, it can be seen that the performance of QWKFDA is affected both by the numbers and the types of the base kernels. The QWKFDA with polynomial kernels possess better performance than those of QWKFDA with Gaussian kernels and sigmoid kernels. The QWKFDA with Gaussian kernels obtains the worst classification accuracy and standard deviation. Table 1 lists the best classification results of QWKFDA method for the three types of base kernels with 10 Gaussian base kernels, 7 polynomial base kernels and with 10 sigmoid base kernels, respectively. The best average classification accuracy of QWKFDA using Gaussian kernel is 92.5% when 10 base kernels are construct weighted kernel function. For the polynomial kernel, it can obtain the highest average classification accuracy, which is 95%, when *n* is 7. The highest average classification accuracy of the sigmoid kernel is also 92.5% when *n* is 10. 

**Figure 4:**
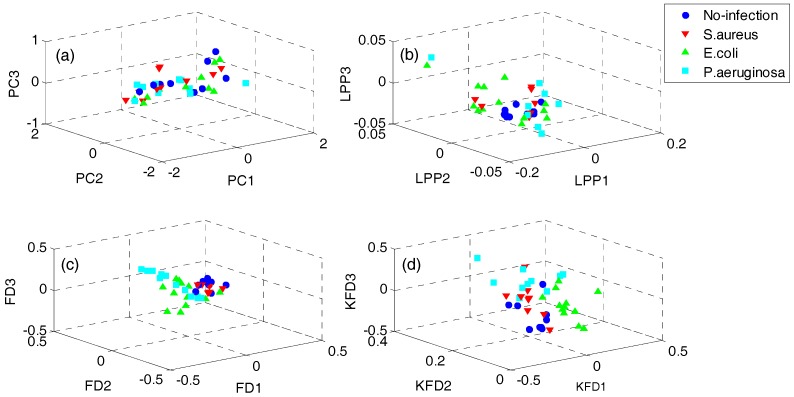
Score plots of (**a**) principal component analysis (PCA), (**b**) locality preserving projections (LPP), (**c**) Fisher discriminant analysis (FDA) and (**d**) kernels Fisher discriminant analysis (KFDA).

**Figure 5:**
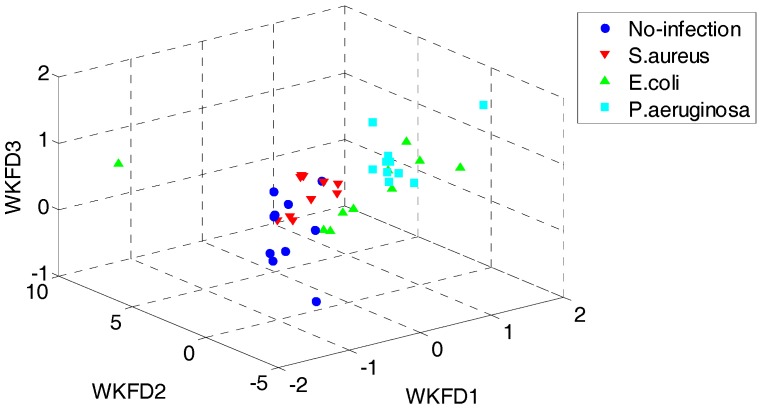
Score plots of QWKFDA.

In order to demonstrate the superiority of the proposed QWKFDA, PCA, LPP, FDA and KFDA are also leveraged as control methods. The number of selected features for all the 5 feature extraction methods is 3. Carry out PCA, LPP, FDA and KFDA for original feature matrix, respectively, and the score plots are demonstrated in Figure 4, where (a–d) are corresponding to PCA, LPP, FDA and KFDA respectively. From Figure 4, the four types of wound samples are not separated wide between classes. Comparing the four sub-graphs, the feature extraction effects based on PCA and LPP are much worse than those of FDA and KFDA, because many more samples are overlapping with the two methods and the samples from the same class are exceedingly scattered. Although some samples are preprocessed by FDA overlap, the within-class samples are very close together. So, it is more likely to get better results than PCA and LPP. KFDA seems more beneficial for recognition than FDA because the samples belong to different classes, which are preprocessed by KFDA, and are farther away from each other than those of FDA. Figure 5 demonstrates the score plots of the proposed QWKFDA. It is obvious that the feature extraction effect based on QWKFDA is much better and four different kinds of samples have been better dispersed. This clear indicates that QWKFDA may possess better pre-classification performance when the number, type and kernel parameters of base kernels are suitably determined.

**Figure 6:**
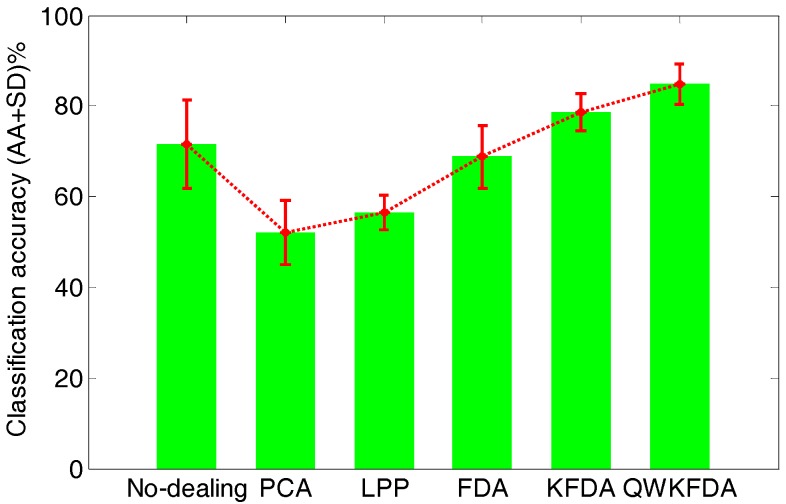
Classification accuracy of different feature extraction methods.

Figure 6 lists the average classification performance of the original feature matrix without any feature extraction preprocessing as well as PCA, LPP, FDA, KFDA and QWKFDA feature extraction methods, which reduce the dimensions of the original features to 3 respectively. The original feature matrix obtains 71.50 ± 9.80% average accuracies. Both PCA and LPP methods have greatly decreased the accuracies of SVM, and the average accuracies are 52.00 ± 7.05% and 56.50 ± 3.94%, respectively. This means that dimension reduction by PCA and LPP will lose much useful information of E-nose data. The classification result of FDA is 68.75 ± 6.90%, which is a bit worse than the original data without being processed by any feature extraction method. The classification result of KFDA, which is 78.75 ± 4.12%, has improved to some extent. It is worth noting that when QWKFDA with a polynomial kernel is used to deal with matrix, much better average accuracy with small standard deviation (84.75 ± 4.48%) is obtained compared with other considered methods.

### 4.2. Results of Dataset II

First of all, the influence of the number of base kernels is evaluated. Table 2 below displays the classification accuracies of QWKFDA with Gaussian kernel, in which the number of base kernels varies from 2 to 10. In Table 2 we can see that the best performance of the QWKFDA method is obtained with 2, 4, and 6 Gaussian base kernels, in which average classification accuracy for the four test batches reaches 93.44% and is the highest among different numbers of base kernels. It is evident that the classification accuracy of batch 5 is much lower than the other batches no matter how many base kernels are used to construct weighted kernel function. This means that although the five batches follow the same system design and implementation, the sensors of batch 5 have severe drift compared with the master batch (batch 1) due to the inherent variability of chemical gas sensors and unknown dynamic processes such as poisoning, aging, or environmental variations.

Secondly, the influence of different types of base kernels is also investigated. Table 3 and Table 4 list the classification results of QWKFDA with polynomial kernels and sigmoid kernels respectively with the numbers of base kernels from 2 to 10. For the polynomial kernel, it can obtain the highest classification accuracy of 90.31% when the number of base kernels is 3, whereas, for the sigmoid kernel, the highest average classification accuracy is 92.19%, which is obtained when 6 base kernels are used to construct the weighted kernel.

From Table 2, Table 3 and Table 4, it can be seen that the performance of QWKFDA is affected both by the numbers and the types of the base kernels. The QWKFDA with Gaussian kernels possess better performance than those of QWKFDA with polynomial kernels and sigmoid kernels. The QWKFDA with polynomial kernels presents better classification accuracy for batch 3 and batch 4, but far lower classification accuracy for batch 5, which leads to the worst average classification accuracy. 

Table 5 lists the average classification performance of the original feature matrix without any feature extraction preprocessing as well as PCA, LPP, FDA, KFDA and QWKFDA feature extraction methods, by which finally 3-dimensional features are selected. It is evidently that QWKFDA with Gaussian kernel can enhance the performance of E-nose a lot compared with other considered methods, as it can choose a more appropriate kernel function to reflect the characteristics of the training data and thus has stronger generalization and robustness.

### 4.3. Discussion

Five different feature extraction methods are employed and compared to capture more useful information of original feature matrixes of the E-noses and improve the classification accuracy. When PCA is used to deal with the original feature, it will worsen the classification performance because PCA is excellent in finding and keeping the linear structure of data and has little to do with nonlinear problems. LPP shares a host of data representation properties of nonlinear techniques. When it is used to deal with E-nose data, the performance of E-nose has worsened too. Because LPP finds the local neighborhood of one point just through computing the distance between points without using the class information, the performance of LPP is dreadful in our study. Classification results of FDA are also not ideal because it obtains the feature matrix by a linear transformation which cannot deal with the nonlinearity of data. In KFDA, nonlinear function is used to transform the nonlinear data of input space to feature space, then FDA is used to deal with the dataset in feature space, and the result proves that kernel function has improved the performance of FDA in extracting the feature information of original feature matrixes. However, its performance is influenced strongly by the kernel function. The performance of the proposed QWKFDA is the most ideal method among the five considered methods. The total classification accuracy is increased a lot for both datasets, and this also proves that the proposed weighted kernel method greatly improves the performance of KFDA. 

## 5. Conclusions

This study mainly investigates a framework of weighted kernel Fisher discrimination analysis for feature extraction of E-nose’s data by introducing multiple kernel methodology, which efficiently combines the advantages of base kernels and constitutes a tradeoff between the base kernels. This approach opens a wide range of further development in the context of Mercer’s kernels for feature extraction of E-nose’s data and provides a new perspective for exploring FDA theory. The results have proved that, as an enhanced KFDA method, the performance of QWKFDA coupled with SVM is much better than those of PCA, LPP, FDA and KFDA on their own. With the help of QPSO, we find the optimal weighted coefficients of the weighted kernel and parameters of the SVM classifier, and finally the performance of an E-nose is enhanced by QWKFDA. The results raised expectations for E-nose as a tool for gas recognition. 

## Figures and Tables

**Table 1:** Confusion matrix of the best classification results of QWKFDA for three types of base kernels.

Class	Predicted as *														
	Gaussian Kernels (n = 10)					Polynomial Kernels (n = 7)					Sigmoid Kernels (n = 10)				
	N	1	2	3	4	N	1	2	3	4	N	1	2	3	4
1	11	**10**	1	0	0	10	**9**	1	0	0	9	**9**	0	0	0
2	10	1	**9**	0	0	10	0	**10**	0	0	8	1	**7**	0	0
3	8	0	0	**7**	1	10	0	0	**10**	0	10	0	0	**9**	1
4	11	0	0	0	**11**	10	0	0	1	**9**	13	0	0	1	**12**
Accuracy	**92.5%**					**95%**					**92.5%**				

* 1, No-infection; 2, *S. aureus*; 3, *E. coli*; 4, *P. aeruginosa*, similarity hereinafter.

**Table 2:** The performance of QWKFDA using Gaussian kernel with the number of base kernels from 2 to 10.

Test Batch	Accuracy Rate (%)								
	2	3	4	5	6	7	8	9	10
Batch 2	99.375	99.375	99.375	99.375	99.375	99.375	99.375	99.375	99.375
Batch 3	99.375	99.375	99.375	99.375	99.375	99.375	99.375	99.375	99.375
Batch 4	100	100	100	98.75	100	98.75	100	97.5	97.5
Batch 5	75	72.5	75	75	75	75	73.75	75	75
Average	**93.44**	92.81	**93.44**	93.13	**93.44**	93.13	93.13	92.81	92.81

**Table 3:** The performance of QWKFDA using polynomial kernel with the number of base kernels from 2 to 10.

Test Batch	Accuracy Rate (%)								
	2	3	4	5	6	7	8	9	10
Batch 2	98.75	98.75	99.375	97.5	98.75	96.875	98.125	96.875	93.75
Batch 3	100	100	100	100	100	100	99.375	100	100
Batch 4	100	100	100	100	100	100	100	100	100
Batch 5	57.5	62.5	52.5	52.5	50	50	52.5	48.75	50
Average	89.06	**90.31**	87.97	87.50	87.19	86.72	87.50	86.41	85.94

**Table 4:** The performance of QWKFDA using sigmoid kernel with the number of base kernels from 2 to 10.

Test Batch	Accuracy Rate (%)								
	2	3	4	5	6	7	8	9	10
Batch 2	98.75	96.875	98.75	96.25	98.75	91.25	91.875	93.125	96.25
Batch 3	96.875	98.75	99.375	100	100	100	100	99.375	100
Batch 4	100	100	100	100	98.75	100	100	100	93.75
Batch 5	71.25	70	70	70	71.25	67.5	72.5	70	68.75
Average	91.72	91.41	92.03	91.56	**92.19**	89.69	91.09	90.63	89.69

**Table 5:** The performance of different control methods.

Test Batch	Accuracy Rate (%)					
	No-Dealing	PCA	LPP	FDA	KFDA	QWKFDA
Batch 2	99.375	95.625	90.625	96.875	93.75	99.375
Batch 3	100	98.125	61.25	71.25	96.875	99.375
Batch 4	97.5	98.75	93.75	91.25	100	100
Batch 5	61.25	61.25	75	62.5	75	75
Average	**89.53**	**88.44**	**80.16**	**80.47**	**91.41**	**93.44**
